# Matrix Gla protein is an independent predictor of both intimal and medial vascular calcification in chronic kidney disease

**DOI:** 10.1038/s41598-020-63013-8

**Published:** 2020-04-20

**Authors:** Armand M. G. Jaminon, Lu Dai, Abdul Rashid Qureshi, Pieter Evenepoel, Jonaz Ripsweden, Magnus Söderberg, Anna Witasp, Hannes Olauson, Leon J. Schurgers, Peter Stenvinkel

**Affiliations:** 1 Department of Biochemistry, Cardiovascular Research Institute Maastricht, Maastricht University, Maastricht, the Netherlands; 2 Division of Renal Medicine, Department of Clinical Science, Technology and Intervention, Karolinska Institutet, Stockholm, Sweden; 3 Department of Immunology and Microbiology, Laboratory of Nephrology, Katholieke Universiteit Leuven, Leuven, Belgium; 4 Division of Medical Imaging and Technology, Department of Clinical Science, Intervention and Technology, Karolinska Institutet, Karolinska University Hospital, Huddinge, Stockholm Sweden; 5 Pathology, Clinical Pharmacology and Safety Sciences, BioPharmaceuticals R&D, AstraZeneca, Gothenburg, Sweden

**Keywords:** Calcification, Chronic kidney disease

## Abstract

Matrix Gla protein (MGP) is a potent inhibitor of vascular calcification (VC) and requires carboxylation by vitamin K to exert calcification inhibition. Chronic kidney disease (CKD) patients undergo early vascular aging often involving extensive VC. The present cross-sectional study investigated the association between circulating dp-ucMGP levels, MGP expression in vascular tissue and MGP polymorphisms. In 141 CKD stage 5 patients, CAC score was significantly increased in the highest tertile of dp-ucMGP (p = 0.002), and a high medial VC score was associated with elevated dp-ucMGP levels. MGP vascular expression was associated with increased circulating dp-ucMGP and CAC scores. MGP SNP analysis revealed that patients homozygous for the C allele of the rs1800801 variant had a higher CAC score (median 15 [range 0–1312]) compared to patients carrying a T allele (median 0 [range 0–966] AU). These results indicate that plasma levels of dp-ucMGP are an independent predictor of increased VC in CKD5 patients and correlate with both higher CAC scores and degree of medial calcification. Additionally, high vascular expression of MGP was associated with higher CAC scores and plasma dp-ucMGP levels. Taken together, our results support that MGP is involved in the pathogenesis of VC.

## Introduction

Matrix Gla protein (MGP) is a vitamin K dependent protein (VKDP) that is involved in the inhibition of vascular calcification (VC). MGP is small secretary protein (14 kD) that is primarily secreted by vascular smooth muscle cells (VSMCs) in the arterial wall^1^. MGP contains five Glu residues that require carboxylation to become activated and to fulfill its calcification inhibitory function. This carboxylation step cannot take place in the absence of vitamin K, which has an unequivocal role in driving this post-translational step^2,3^. Vitamin K is a co-factor for the enzyme γ-glutamyl carboxylase that converts glutamic acid (Glu) into γ-carboxyglutamic acid (Gla) residues^2^. This conversion is critical for the activation of MGP. Additionally, there are three serine residues that need phosphorylation^4,5^. The exact role of phosphorylation of MGP is still not known, but it is believed to play an important role in the regulation of secretion of the protein^3^. Upon activation, MGP binds calcium-salts with high affinity, thereby affecting the calcification processes. The importance of MGP in the inhibition of calcification is illustrated by studies of MGP knockout mice, who die within two months after birth due to severe arterial calcification and rupture of the aorta^1^.

Chronic kidney disease (CKD) patients have an extremely high risk for developing vascular disease^4^. VC, manifested both as medial and intimal calcification with distinct pathologies, is a common risk factor in CKD^5^. Additionally, vitamin K deficiency is frequently encountered in CKD, which is associated with increased plasma levels of dephosphorylated uncarboxylated MGP (dp-ucMGP) plasma levels^6,7^. Furthermore, increased plasma dp-ucMGP levels associate with VC^8,9^, cardiovascular morbidity-mortality^10^ and aortic valve calcification^11^. However, the connection between local vessel wall expression of MGP (transcription), production (tissue ucMGP) and excretion (plasma dp-ucMGP) is not well explored in CKD. In addition, the vasculature is exposed to a toxic uremic milieu, which has been shown to affect carboxylase activity causing vascular vitamin K deficiency and increased VC^12^. Additionally, uremia promote VC in a rat model^13^ and induced bone-specific proteins in cultured VSMCs^14^.

The use of dp-ucMGP has clinical potential as a prognostic biomarker of VC^15^ and might provide complementary information to traditional cardiovascular disease (CVD) risk factors^16^. Furthermore, carboxylated MGP bind, via the negative charge, to active calcification. The inactive form of MGP (dp-ucMGP) is set free in the circulation because of lack of negative charge to bind to calcium crystals and, thus, might be used as biomarker to identify high-risk CVD patients, allowing early intervention^17^. Furthermore, although single nucleotide polymorphisms (SNP) of MGP have been associated with outcomes in diabetes and CKD, its association to VC remains obscure. MGP SNP analysis could help us understand the complex nature of MGP expression and regulation. Other VKDPs, such as osteocalcin (OC) share the carboxylation step to become activated. Similar to MGP, uncarboxylated OC (ucOC) is increased in vitamin K deficiency^18^ and has been reported to have a role in the development of VC^19^.

We investigated the potential association between MGP and VC in CKD stage 5 patients undergoing living donor renal transplantation (RTx). We link MGP genetics (SNPs), transcription (mRNA) and protein data (immunohistochemistry and plasma levels) to clinical vascular phenotype (CAC and histology).

## Results

### Clinical and biochemical characteristics

Demographic and clinical characteristics are shown in Table 1 and Supplemental Table S1. Patients in the highest tertile of dp-ucMGP (>1491 pM) levels were older and had higher CVD prevalence (25%). Moreover, serum creatinine was higher in the highest tertile of dp-ucMGP compared to the other two tertiles (782 μmol/L). Additionally, glu-OC (37.8 ng/mL), mid-OC (90.5 ng/mL) and Osteoprotegerin (7.6 pg/mL) were also elevated in the highest tertile of dp-ucMGP.

**Table 1:** Baseline clinical and biochemical characteristics in 141 CKD 5 patients in relation to tertiles of dp-ucMGP.

	All (n = 141)	1^st^ tertile (n = 46)	2^nd^ tertile (n = 47)	3^rd^ tertile (n = 48)	p-value
Age (years)	47 (24, 63)	43 (22, 63)	49 (23, 62)	50 (32, 68)	0.008
Males, (%)	70	65	68	75	0.57
Diabetes mellitus, (%)	11	7	11	17	0.29
Cardiovascular disease, (%)	16	7	15	25	0.04
BMI, (kg/m^2^)*	24.5 (20.8, 29.9)	24.0 (19.8, 29.1)	23.5 (20.8, 30.4)	25.1 (21.8, 30.2)	0.17
Systolic BP, (mmHg)*	140 (117, 169)	140 (116, 162)	140 (116, 167)	144 (121, 181)	0.29
Diastolic BP, (mmHg)*	84 (69, 96)	82 (68, 92)	82 (72, 100)	86 (67, 99)	0.33
Hemoglobin, (g/L)‡	114 (99, 132)	111 (98, 129)	118 (99, 135)	113 (99, 132)	0.09
HbA1c, (mmol/mol)†	33 (23, 40)	35 (26, 40)	32 (25, 40)	32 (21, 41)	0.06
Triglycerides, (mmol/L)	1.3 (0.7, 2.4)	1.2 (0.6, 2.2)	1.2 (0.7, 2.6)	1.4 (0.7, 2.6)	0.58
Total cholesterol, (mmol/L)	4.5 (3.1, 6.2)	4.3 (3.0, 6.1)	4.6 (3.2, 6.0)	4.5 (3.0, 6.4)	0.64
HDL cholesterol, (mmol/L)	1.4 (0.9, 2.1)	1.4 (1.0, 2.1)	1.4 (0.9, 2.0)	1.3 (0.8, 2.2)	0.37
Serum Creatinine, (µmol/L)	723 (488, 1022)	622 (388, 985)	743 (517, 1018)	782 (531, 1135)	0.01
Serum Albumin (g/L)*	35 (29, 40)	35 (30, 42)	35 (29, 40)	36 (29, 41)	0.80
Uric Acid, (µmol/L)•	369 (254, 522)	413 (285, 557)	350 (239, 510)	357 (247, 469)	0.02
PEW (SGA > 1), n (%)**	25	31	26	18	0.33
**Vascular calcification biomarkers**					
Coronary artery calcium, (AU) ‡‡	8 (0, 1170)	0 (0, 236)	8 (0, 1392)	34 (0, 1948)	0.002
Calcium, (mmol/L)*	2.3 (2.0, 2.5)	2.3 (2.1, 2.4)	2.3 (2.0, 2.6)	2.3 (1.9, 2.6)	0.67
Phosphate, (mmol/L)*	1.6 (1.0, 2.4)	1.7 (1.0, 2.2)	1.6 (1.1, 2.5)	1.7 (0.9, 2.5)	0.96
Glu-OC, (ng/mL)**	14.0 (1.8, 87.6)	7.5 (1.6, 33.7)	15.5 (1.7, 122.6)	37.8 (2.3, 99.2)	0.001
Gla-OC, (ng/mL)**	33.2 (11.7, 97.6)	26.7 (11.0, 73.5)	33.2 (11.7, 138.0)	42.9 (9.7, 102.2)	0.23
Mid-OC¸ (ng/mL) ¶¶¶	59.5 (13.5, 259.5)	37.9 (11.6, 178.6)	61.8 (11.3, 218.6)	90.5 (17.7, 395.8)	0.02
Osteoprotegerin, (pg/ml) •••	6.1 (3.8, 12.0)	5.6 (2.8, 10.1)	5.9 (3.9, 11.1)	7.6 (4.2, 14.0)	0.002

Data presented as median (10th – 90th percentile) or percentage.

**Abbreviations:** CKD, chronic kidney disease; dp-ucMGP, dephosphorylated uncarboxylated Matrix Gla protein; BMI (body mass index); BP, blood pressure; HbA1c, Hemoglobin A1c; HDL, high-density lipoprotein; PEW, protein energy wasting; SGA, subjective global assessment; AU, Agatson unit; Glu-OC, undercarboxylated osteocalcin; Gla-OC, carboxylated osteocalcin; mid-OC, n-mid osteocalcin.

*n= 139 **n= 137 ***n= 109.

^‡^*n*= 120 ^‡‡^*n*= 110 ^‡‡‡^*n*= 103.

^†^*n*= 121 ^††^*n*= 99 ^†††^*n*= 68.

^¶^*n*= 106 ^¶¶^*n*= 138 ^¶¶¶^*n*= 91.

^•^*n*= 128 ^••^*n*= 140 ^•••^*n*= 114.

### dp-ucMGP levels were associated with increased CAC and medial calcification score

**Figure 1:**
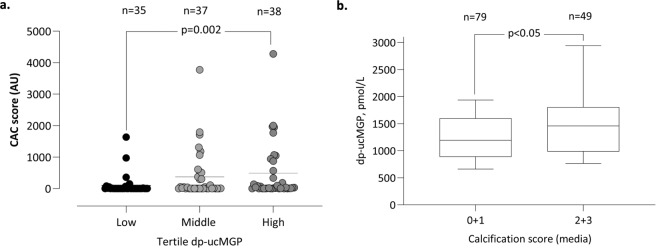
dp-ucMGP levels associate to CAC score and vascular media calcification score. (**a)** CAC score measured in 110 CKD5 patients in relation to tertiles of dp-ucMGP levels. **(b)** dp-ucMGP levels of 128 CKD5 patients displayed against low (score 0 + 1) and high (score 2 + 3) media VC.

Patients in the 3^rd^ tertile showed higher CAC score compared to the other two tertiles ranging from a CAC score with a median (10^th^ – 90^th^ percentile) of 0 (0, 236) in the 1^st^ 8 (8, 1392) in the 2^nd^ and 34 (0, 1948) in the 3^rd^ dp-ucMGP tertile (p = 0.002) (Fig. 1a). Medial VC (scored by pathologists, ranging from 0 - no, 1 – mild, 2 – moderate, to 3 - severe calcification) was associated with an increase in dp-ucMGP levels (median 1193 in group 0–1 and 1458 in group 2–3) (p < 0.05) (Fig. 1b).

### Calcification of the vascular media co-localized with ucMGP

**Figure 2:**
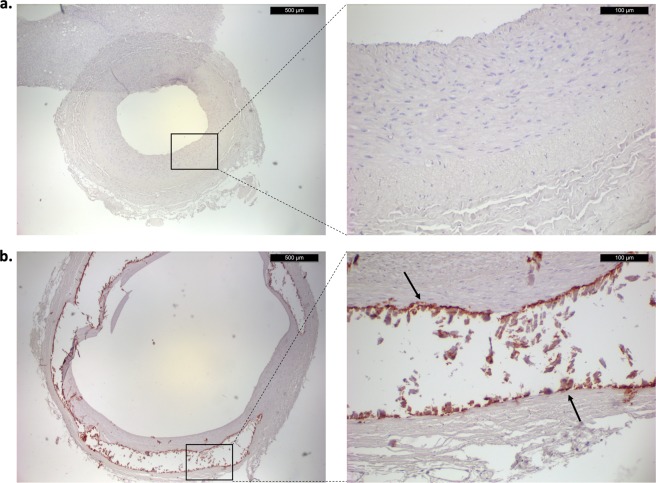
Immunohistochemical staining of ucMGP in epigastric artery from CKD5 patients. Panel a shows ucMGP staining of epigastric artery of a patient with a medial VC score of 0 (left, 4×; right, 20×). Panel b shows ucMGP staining of an epigastric artery with a medial VC score of 3 (left, 4×; right, 20×).

Patients that had a medial VC score of 0 showed no staining for ucMGP (Fig. 2a). On the contrary, patients that had a medial VC score of 3 showed a strong staining of ucMGP around calcified areas (Fig. 2b). Calcified areas are accompanied by a loss of structure and show no cellular components, the arrows indicate calcified areas that stain highly positive for ucMGP (Fig. 2b).

### Multivariate analysis of factors associated with dp-ucMGP

In multivariate linear regression analysis of determinants, bone markers were associated with dp-ucMGP (i.e. 1-SD increase) including ucOC (est 0.48; p = 0.001) and OPG (est 0.28; p = 0.02) after adjusting for 1-SD increase of age and albumin (Table 2).

**Table 2:** Multiple linear regression models for 1-standard deviation increase of plasma dp-ucMGP in 141 CKD 5 patients.

	Model (coefficient + SE, p-value)
1-SD increase of age, years	0.05 + 0.12 (0.69)
1-SD increase of ucOC, ng/ml	0.48 + 0.14 (0.001)
1-SD increase of albumin, g/L	0.13 + 0.12 (0.27)
1-SD increase of Mid-OC, ng/ml	−0.21 + 0.15 (0.16)
1-SD increase of osteoprotegerin, pg/ml	0.28 + 0.12 (0.02)

**Abbreviations**; 1-SD, one standard deviation; dp-ucMGP, dephosphorylated uncarboxylated Matrix Gla protein; CKD, chronic kidney disease; SE, standard error; ucOC, undercarboxylated osteocalcin; mid-OC, n-mid osteocalcin.

### Multivariate analysis of factors associated with medial calcification

In a multivariate logistic regression analysis of factors associated with high medial VC (Table 3) (VC score media (0–1) = reference), high medial VC was associated with age (p = 0.006), sex (p = 0.001), dp-ucMGP (p = 0.04) and DM (p = 0.008) after adjusting for hsCRP and CAC score (p = 0.059) (Table 3).

**Table 3:** Multinominal logistic regression analysis of factors associated with high calcification score media (2 + 3) (n = 122, pseudo r^2^ = 0.22). Patients with calcification score media 0 + 1 served as the reference.

	High calcification score media (Q2 + Q3)	
	Odds ratio (95%CI)	p-value
1-SD increase of age, years	2.3 (1.3–4.1)	0.006
1-SD increase of dp-ucMGP, pmol/L	1.6 (1.0–2.5)	0.04
Gender (female, male)	7.3 (2.2–25.1)	0.001
1-SD increase of hsCRP, mg/L	1.3 (0.4–1.3)	0.31
Diabetes mellitus (yes, no)	26.0 (2.3–293.2)	0.008
1-SD increase of CAC-score, AU	1.2 (0.5–2.9)	0.59

**Abbreviations:** 95%CI, 95% confidence interval; 1- SD, one standard deviation; dp-ucMGP, dephosphorylated uncarboxylated Matrix Gla protein; hsCRP, high-sensitivity C-reactive protein; CAC, coronary artery calcium; AU, Agatston unit.

### High arterial *MGP* mRNA expression associates with high CAC

**Figure 3:**
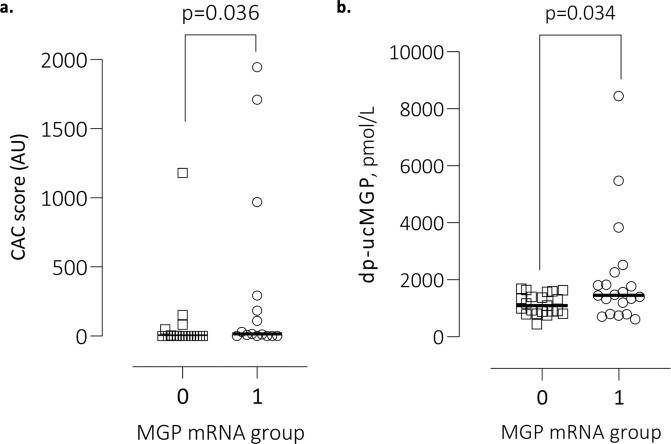
Association between MGP expression and CAC and dp-ucMGP. (**a**) MGP expression measured in 41 CKD5 patients in relation to CAC score. **(b)** MGP expression measured in 41 CKD5D patients in relation to dp-ucMGP plasma levels.

We divided patients according to median of MGP mRNA expression. High expression of MGP (n = 41) showed higher CAC score with a median (10^th^ – 90^th^ percentile) of 16 (0–1804), compared to the low MGP expression group with a median (10^th^ – 90^th^ percentile) of 0 (0–255) (p = 0.036) (Fig. 3a). Additionally, high expression of MGP showed higher dp-ucMGP plasma levels, with a median (10^th^ - 90^th^ percentile) of 1459 (711–5313), compared to low MGP expression with a median (10^th^ - 90^th^ percentile) of 1100 (760–1653) (p = 0.034) (Fig. 3b). In a Spearman’s rank (rho) analysis, MGP expression was significantly correlated with CAC score (Rho = 0.39) and medial calcification score (Rho = 0.37). After adjusting for age and sex in a multivariate linear regression analysis, these significant correlations were lost.

### MGP polymorphisms - relation to circulating dp-ucMGP, arterial MGP mRNA and VC

Distributions of MGP rs4236, rs1800801, rs1800802 polymorphisms in CKD5 patients and controls are presented in Supplemental Table S2. The genotype frequencies of the rs4236 and rs1800801, but not rs1800802, differed significantly between patients and controls: in patients, the rs4236 T/T and rs1800801 C/C genotypes were more common (44% *vs*. 39% and 53% *vs*. 44%, respectively), whereas rs4236 C/C and rs1800801 T/T were less frequent (both 6% *vs*. 15%) compared to controls. A selection of patient characteristics was investigated in relation to MGP genotypes (Supplemental Table S3). None of the MGP SNPs were associated with age, sex, DM or dp-ucMGP levels. Neither the rs1800801 nor the rs1800802 SNPs associated with *MGP* mRNA levels but when dividing patients according to rs4236 T-homozygotes *vs*. C-allele carriers, *MGP* mRNA levels were found to be higher in patients homozygous for the T allele compared to patients with CC and CT genotype (1.3 *vs*. 0.9 RQ, p = 0.03). Whereas patient’s CAC scores were not associated with MGP genotypes, media VC score differed significantly according to rs1800801 genotype, with 50% of C homozygotes having a moderate to extensive calcification (i.e. score 2 or 3) compared to 24% and 14%, respectively, in patients with C/T and T/T genotypes (p = 0.01) (Supplemental Table S3). When comparing C-allele homozygotes to T-allele carriers in relation to either none/minimal calcification (score 0–1) or moderate/extensive calcification (score 2–3) the difference became even more significant (50% *vs*. 22%, n = 109; p = 0.005). Similarly, patients that were homozygous for the rs4236 T-allele had more often moderate-extensive medial VC than patients carrying a C-allele (49% *vs*. 27%, p = 0.02).

## Discussion

We examined the role of MGP as a potential biomarker for the extent of VC in CKD5 patients and confirmed that an increase in plasma dp-ucMGP levels was significantly associated with an increase in both CAC score and medial arterial VC. CKD patients are predisposed to VC^20^, an independent risk factor for CVD morbidity and mortality^4,21^. CAC score determines the extent of CAC and is correlated to VC at other places in the arterial tree^22–24^. However, as CT of the heart involves radiation and its value to determine microcalcifications is limited, circulating biomarkers that reflect early VC would be cheaper, safer and less invasive. Additionally, to interfere with VC progression, biomarkers that predict initiation phases of VC, such as MGP are of value in prediction and to follow treatment of VC.

MGP is a potent inhibitor of VC produced by VSMCs^25^, which are pivotal in maintaining MGP homeostasis, as they are involved in both the production and activation of MGP, both of which are important for the subsequent inhibition of VC^21,25^. Since MGP is an active inhibitor of VC it could be a potential useful biomarker. Indeed, circulating plasma levels of dp-ucMGP correlate with amount of VC^22,23^ and arterial stiffness^26^. In accordance, we report that patients with the highest dp-ucMGP levels had a higher CAC score and more extensive medial VC.

CKD patients suffer from vitamin K deficiency and are prone to develop VC^24^. Since vitamin K is necessary for the activation (carboxylation) of MGP in the vessel wall, MGP will be produced in the inactive form when levels of vitamin K are low^25^. We show that next to circulating levels of dp-ucMGP, highly calcified epigastric arteries also show co-localization with ucMGP. This data supports the hypothesis that circulating dp-ucMGP reflects local tissue levels of ucMGP which might be caused by a vascular vitamin K deficiency, a risk factor for VC. Although vitamin K intake or plasma levels were not measured in the present study, our findings indirectly imply that vitamin K status is low since also ucOC was significantly increased^27^. After correction for age and albumin, dp-ucMGP remained significant for bone markers. Moreover, after correction for CAC score and inflammation, dp-ucMGP remained significant for medial calcification score.

In this study, high MGP expression was associated with CAC score and increased medial calcification (not significant) scored by pathologists. High expression of MGP infers to a high local production of MGP. However, nothing is known about the activation state of this locally produced MGP. As CKD patients are vitamin K deficient^24^, we hypothesize that most of the MGP is present as inactive MGP. This accord with our results showing a strong association between MGP expression and circulating dp-ucMGP. *In vitro*, low expression of MGP was associated with calcification via upregulation of BMP-2^28^. Additionally, under specific pro-calcific conditions, such as a high calcium concentration, calcification is increased whereas MGP expression is decreased^29^. This is contradictory to our results but might be explained by the carboxylation efficiency of MGP. Total MGP in the plasma is the sum of both active (cMGP) and inactive (ucMGP) MGP; thus, high levels of dp-ucMGP do not reflect the total level of MGP that is expressed in the tissue. The importance of MGP as inhibitor of VC is demonstrated by MGP *ko* studies in mice that showed increased soft tissue calcification^30^. Moreover, MGP^-/-^ mice express higher levels of osteogenic proteins, such as osteopontin, OC and Cbfa1^31^. Due to the complex nature of MGP, further studies need to examine the precise relation between local vasculature MGP expression and circulating MGP.

Besides measurement of plasma dp-ucMGP, we genotyped a subgroup of patients for three well-known SNPs (rs4236, rs1800801 and rs1800802) in the MGP gene. In comparison to healthy controls, our subgroup of CKD patients differed in respect to two of the SNPs, rs4236 and rs1800801, with patients displaying clearly lower frequencies of the minor alleles C and T, respectively. As none of the SNPs were associated with plasma dp-ucMGP levels, the biological impact is unclear. However, because the MGP rs1800801 T-allele is associated with lower medial VC score, this allele may be protective. The literature is, however, conflicting regarding the potential protective effect of the T-allele on vascular disease. A meta-analysis by Sheng *et al*.^32^ showed that the MGP T-allele was associated with a higher risk for VC whereas Taylor *et al*.^33^ found no association between the MGP rs1800801 and CAC score. An explanation for the conflicting evidence might be the differences in ethnic composition between the study populations and limited power.

The present results should be considered in light of some strengths and limitations. The relatively low sample size restricts power of the multivariate analyses and information retrieved from the MGP gene variants. Moreover, the observational nature of the study restricts causal conclusions. Furthermore, results obtained in this study are retrieved from different patient cohorts which means that our study population is heterogenous, for example displaying a population aged from 19–75. Since dp-ucMGP only represents a minor fraction of the total ucMGP content it should be interpreted with care^17^. CAC determination by cardiac CT cannot distinguish between medial or intimal calcification. However, we did find a strong association between medial VC in the epigastric artery and CAC. Finally, expression levels of MGP were only measured in limited number of patients and further studies are needed to examine the role of MGP expression in VC. For the MGP gene variants we need to perform additional *in vitro* and *in vivo* experiments to assess its causal role.

As increased dp-ucMGP levels associate with increased CAC and medial VC our results support that dp-ucMGP is an independent predictor of VC and a risk factor for arterial stiffness and cardiovascular mortality^34^. Alterations in plasma dp-ucMGP correlate with local tissue expression of ucMGP around areas of VC, high general MGP expression and genomic SNP analysis. Although our data indicate a predictive value of MGP as a biomarker for VC, further studies need to confirm whether these finding translate into CVD morbidity and mortality. Additionally, discovering the precise regulation of MGP in VC may provide novel therapeutic approaches with a potential role for vitamin K supplementation.

## Materials and methods

### Patients and study design

Adult ESRD patients undergoing living donor (LD) kidney transplantation (tx) at the Department of Transplantation Surgery at Karolinska University Hospital between March 2009 and October 2016 were invited to participate in the study. The etiologies of CKD were chronic glomerulonephritis (n = 52), hypertension and renovascular disease (n = 8), diabetic nephropathy (n = 9) and others or unknown causes (n = 72). The cohort included CKD5 non-dialysis (ND) patients (n = 51), prevalent peritoneal dialysis (PD) patients (n = 39) and prevalent hemodialysis (HD) patients (n = 51). PD patients were treated (median vintage time 11.4 months) with different combinations of biocompatible glucose-based or amino acid-based, or, for the long dwell, icodextrin-based solutions. HD-patients were treated by conventional maintenance HD or other dialytic techniques such as hemodiafiltration (median vintage time 14.4 months). Sixteen (11%) out of 141 patients had diabetes. Twenty-three (15%) of the patients had previously been diagnosed with cerebrovascular, cardiovascular, and/or peripheral vascular disease (grouped as CVD). We measured circulating plasma dp-ucMGP in 141 CKD5 patients including 51 non-dialyzed (CKD5-ND) and 90 CKD5 patients treated by either PD (n = 39) or HD (n = 51). Patient characteristics are shown in Table 1 and a flow chart of patient inclusion is displayed in Fig. S1. Age ranged from 19 to 75 years and patients were recruited from March 2009 to October 2016.

Exclusion criterion was unwillingness to participate. Informed consent was obtained from each patient. The Ethics Committee of the Karolinska Institutet approved study protocols. The studies were conducted in adherence to the Declaration of Helsinki.”

### Biochemical assessments

Prior to the LD-RTx, fasting blood samples were drawn and stored in −80 °C. Biochemical analyses of plasma cholesterol, triglycerides, HDL-cholesterol, hemoglobin, creatinine, calcium, phosphate, albumin (coefficient of variation, CV, 3–4%), were performed at the Clinical Chemical Laboratory of Karolinska University Hospital, Stockholm, Sweden. LDL was calculated using the Friedewald formula: [(total cholesterol) - (high-density lipoprotein cholesterol) – (triglycerides/5)].

### Plasma dp-ucMGP

Plasma dp-ucMGP levels were determined using the commercially available IVD CE-marked chemiluminescent InaKtif MGP assay on the IDS-iSYS system (IDS, Boldon, UK). In brief, patient samples and internal calibrators were incubated with magnetic particles coated with murine monoclonal antibodies against dp-MGP, acridinium-labelled murine monoclonal antibodies against ucMGP, and an assay buffer. The magnetic particles were captured using a magnet and washed to remove any unbound analyte. Trigger reagents were added; the resulting light emitted by the acridinium label was directly proportional to the level of dp-ucMGP in the sample. The within-run and total variations of this assay were 0.8–6.2% and 3.0–8.2%, respectively. The assay measuring range was between 300 and 12,000 pmol/L and was linear up to 11,651 pmol/L. Assays were performed in a single run by Coagulation Profile BV, department of Biochemistry, Maastricht, the Netherlands.

### Vascular scoring by histology

Inferior epigastric arteries (n = 118) were collected from patients within 20 min from the start of kidney transplantation procedure. After fixation (4% phosphate buffered formalin) and paraffin embedding, 1–2 μm thick tissue sections were stained with hematoxylin, eosin and von Kossa method before evaluation by experienced pathologists. The extent of medial calcification was assessed by a pathologist in vascular biopsies^35^ and graded as 0 to 3. Patients graded as 0 and 1 represented no/minimal vascular calcification, and those graded 2 and 3 represented moderate/extensive vascular calcification.

### ucMGP immunohistochemical staining

After deparaffinization and rehydration of inferior epigastric arteries (n = 20), immunostaining for ucMGP was performed as described previously^36^, using a monoclonal MGP antibody directed against uncarboxylated MGP (1 mg/mL, 1:400 diluted) and a goat anti-mouse HRP (60 minutes at room temperature; Dako, Golstrup, Denmark) secondary antibody. Detection was performed using Novared stain (Vector Labs, Burlingame, CA), yielding a red color. The degree of ucMGP staining was measured semi-quantitatively and assessed by independent pathologists and graded as 0 to 3. Patients graded as 0 and 1 represented zero to minimal ucMGP staining, and patients graded 2 and 3 represented moderate to extensive ucMGP positivity.

### *MGP* expression

Inferior epigastric artery samples collected at LD-RTx (n = 41) were incubated overnight in AllProtect Tissue Reagent (Qiagen, Hilden, Germany) and subsequently stored at −70 °C. RNA was isolated from arteries using TRIzol Reagent and *MGP* mRNA levels were analysed with TaqMan chemistry (Thermo Fisher Scientific, Waltham, MA USA) as previously described^37^.

### Genotyping of MGP rs4236, rs1800801 and rs1800802

A subset of 117 CKD5 patients with available peripheral blood samples and 389 controls (anonymous blood donors recruited in Stockholm municipality) underwent genotyping. Genomic DNA was extracted following standard procedures at the Karolinska Biobank. Genotypes for rs4236, rs1800801 and rs1800802 polymorphisms were determined with PCR-based allelic discrimination using TaqMan SNP Genotyping Assays, QuantStudio 5 System (Thermo Fisher Scientific, Waltham, MA, USA) and TaqMan Genotyper Software according to manufacturer’s protocols.

### CAC score

Cardiac computed tomography (CT) scans were performed using a 64-channel detector scanner (Lightspeed VCT; General Electric (GE) Healthcare, Milwaukee, WI). CAC scores were expressed in Agatston units^38^, the protocol and measurements as described previously in detail^38^. Total CAC score was calculated as the sum of CAC scores in the left main artery, the left anterior descending artery, the left circumflex artery, and the right coronary artery.

### Statistical analyses

Continuous data are expressed as median (10th to 90th percentile) and nominal or ordinal data as percentage. Statistical significance was set at the level of P < 0.05. Comparisons between groups were assessed with the non-parametric Wilcoxon test/Kruskal-Wallis ANOVA-test for continuous variables and Chi-square test for nominal variables. Non-parametric Spearman rank correlation analysis was used to determine associations between variables. We performed multiple imputation of missing values for multivariate linear regression and multivariate logistic regression using the function PROC MI, with all variables in the covariate section used to produce the values for imputation. The original *n* for each variable is given throughout. The results for each imputation were generated using PROC REGRESS and LOGISTIC, and then combined using PROC MIANALYZE. We used 20 imputed datasets for this study to ensure that our effect estimates were not overly inaccurate due to Monte Carlo variability. Multivariate linear regression analyses of dp-ucMGP were used and results were shown as standardized β regression coefficients. We performed multinomial logistic regression analysis to examine factors associated for determinants of vascular calcification 0–1 score vs 2–3. Statistical analyses were performed using statistical software SAS version 9.4 (SAS Campus Drive, Cary, NC, USA) and Stata 15.1 (Stata Corporation, College Station, TX, USA)^38^. Figures were created using GraphPad Prism version 8.3.1 for Windows, GraphPad Software, San Diego, California USA, www.graphpad.com.

## Supplementary information

Supplementary material.
