# Supplementary material for: Matrix Gla protein is an independent predictor of both intimal and medial vascular calcification in chronic kidney disease
Source: Sci Rep. 2020 Apr 20;10:6586. doi: 10.1038/s41598-020-63013-8 (PMC7171129; doi:10.1038/s41598-020-63013-8)
Supplement: Supplementary file 1 — Supplementary material. [file 41598_2020_63013_MOESM1_ESM.pdf]

Matrix Gla protein is an independent predictor of both intimal and medial vascular calcification in chronic kidney disease

Armand M.G Jaminon[1], Lu Dai[2], Abdul Rashid Qureshi[2], Pieter Evenepoel[3], Jonaz Ripsweden[4], Magnus Söderberg[5], Anna Witasp[2], Hannes Olauson[2], Leon J. Schurgers[1+], Peter Stenvinkel[2+*]

1. Department of Biochemistry, Cardiovascular Research Institute Maastricht, Maastricht University, the Netherlands
2. Division of Renal Medicine, Department of Clinical Science, Technology and Intervention, Karolinska Institutet, Stockholm, Sweden
3. Department of Immunology and Microbiology, Laboratory of Nephrology, Katholieke Universiteit Leuven, Leuven, Belgium.
4. Division of Medical Imaging and Technology, Department of Clinical Science, Intervention and Technology, Karolinska Institutet, Karolinska University Hospital, Huddinge, Stockholm, Sweden.
5. Pathology, Clinical Pharmacology and Safety Sciences, BioPharmaceuticals R&D, AstraZeneca, Gothenburg, Sweden.

[+]Contributed equally to present work
[*]Corresponding author

Correspondence: peter.stenvinkel@ki.se
Peter Stenvinkel, FERA, FASN
Professor of Renal Medicine
Division of Renal Medicine M99
Dept of Clinical Science Technology and Intervention
Karolinska Institutet
Stockholm
Sweden
Phone +4673-6998771

Supplementary information

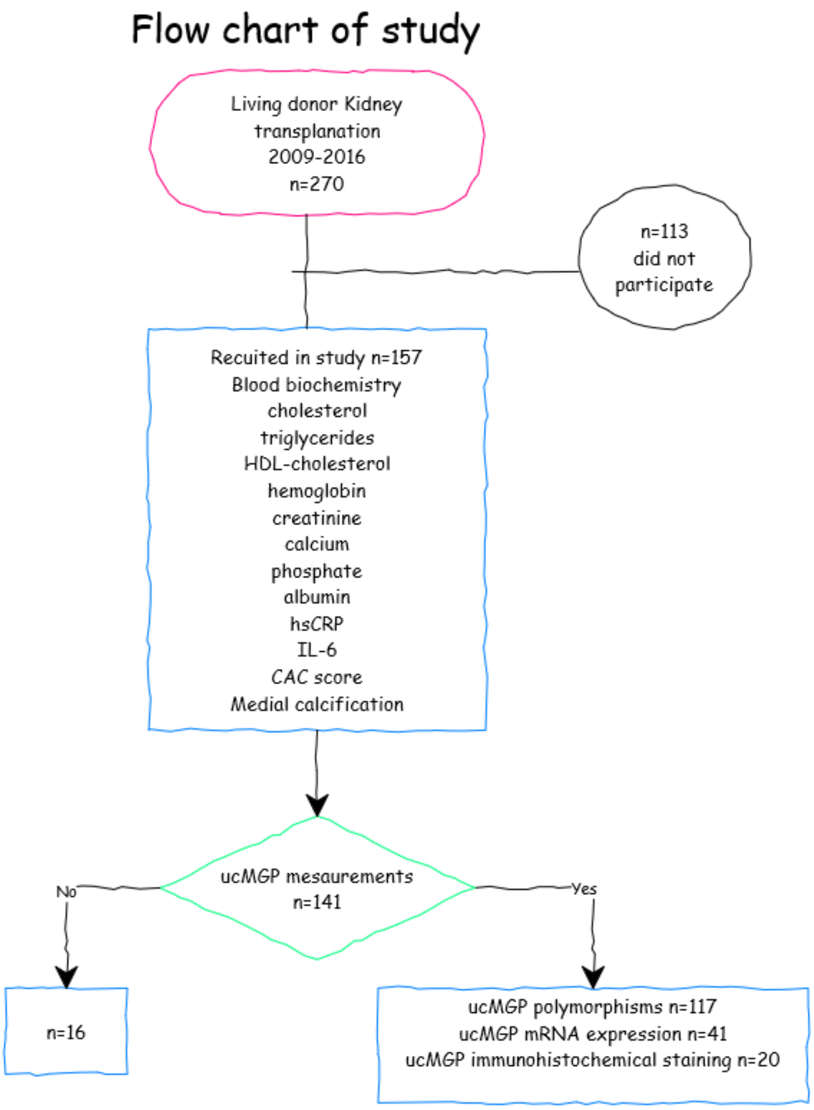

Supplemental Figure S1 flow chart of patient inclusion

Supplemental Table S1 Other baseline clinical and biochemical characteristics in 141 CKD 5 patients in relation to tertiles of dp-ucMGP

	All (n=141)	1st tertile (n=46)	2nd tertile (n=47)	3rd tertile (n=48)	p-value
iPTH, (ng/L)	261 (83, 558)	255 (81, 747)	270 (84, 502)	290 (49, 563)	0.72
1,25-OH vitamin, (mmol/L) ††	18 (9, 32)	18 (11, 35)	18 (9, 33)	17 (7, 32)	0.89
25(OH) D-vitamin, (mmol/L) ¶¶	35 (17, 72)	33 (16, 75)	38 (12, 69)	36 (21, 73)	0.64
hsCRP, (mg/L) ••	0.7 (0.2, 4.0)	0.5 (0.2, 2.5)	0.7 (0.2, 3.9)	1.5 (0.3, 10.7)	<0.001
IL-6, (pg/mL) ***	1.3 (0.1, 4.1)	0.9 (0.01, 3.0)	1.6 (0.3, 3.4)	1.4 (0.02, 6.6)	0.06
TNF, (pg/mL) ‡‡‡	10.5 (7.6, 16.5)	8.9 (7.3, 12.3)	10.7 (7.3, 16.1)	12.6 (9.1, 20.4)	<0.001
CTX, (ng/ml) •	1.8 (0.5, 5.1)	1.8 (0.4, 4.9)	1.8 (0.6, 5.5)	1.9 (0.5, 4.4)	0.88
FGF-19, (pg/ml) *	117 (41, 331)	133 (39, 439)	86 (35, 263)	135 (53, 343)	0.02
FGF-23, ††† (pg/ml)	4055 (676, 48027)	3668 (350, 32935)	7581 (826, 44887)	6391 (624, 99267)	0.40
Klotho, (pg/ml) ••	389 (213, 669)	366 (188, 763)	382 (212, 671)	414 (257, 585)	0.66
ALP, (U/L)	65.9 (39.8, 118.3)	69.7 (41.7, 118.2)	65.1 (37.4, 122.9)	65.5 (39.4, 121.5)	0.95
BALP, (ug/L)	15,8 (8.6, 41.5)	16.1 (8.9, 40.7)	14.7 (8.6, 41.1)	17.2 (8.3, 43.4)	0.84
Sclerostin, (pg/ml) **	432 (245, 830)	409 (215, 802)	440 (244, 807)	431 (259, 897)	0.55
		**Medication**			
Statins, (%)	37	30	36	44	0.41
β-blockers, (%)	58	44	62	69	0.04
Calcium blocker, (%)	51	54	55	44	0.46
Phosphate binders, (%)	53	54	53	52	0.98

Data presented as median (10th – 90th percentile) or percentage

Supplemental Table S2 Genotype distributions of *MGP* polymorphisms rs4236, rs1800801 and rs1800802 in 117 CKD5 patients and 389 controls.

		CKD5 patients	Controls	P-value*
**MGP rs4236, n (%)**	**T/T**	51 (44)	152 (39)	0.03
	**T/C**	59 (50)	177 (46)	
	**C/C**	7 (6)	60 (15)	
**MGP rs1800801, n (%)**	**C/C**	62 (53)	171 (44)	0.03
	**C/T**	48 (41)	161 (41)	
	**T/T**	7 (6)	57 (15)	
**MGP rs1800802, n (%)**	**A/A**	69 (59)	232 (59)	0.89
	**A/G**	42 (36)	145 (37)	
	**G/G**	6 (5)	16 (4)	

*Pearson's Chi-Square test

Supplemental Table S3 Distribution of patient characteristics in relation to *MGP* genotypes in 117 CKD5 patients.

		***MGP* genotypes**			**P-value**
**rs4236**					
		**T/T**	**T/C**	**C/C**	
Age (years)		47 (24-64)	45 (23-62)	39 (28-66)	0.58
Males (%)		76	63	43	0.11
DM (%)		10	8	14	0.88
dp-ucMGP (pmol/L)		1333 (745-2218)	1193 (661-2526)	1329 (799-1998)	0.65
MGP mRNA (RQ)≠		1.2 (0.3-3.4)	0.9 (0.4-1.7)	0.61 (0.5-0.8)	0.15
CAC score (AU)#		37 (0-1729)	1 (0-1068)	0 (0-12)	0.09
Media VC score, (%)¤	0	11	18	29	0.30
	1	40	54	57	
	2	31	20	0	
	3	18	9	14	
rs1800801					
		**C/C**	**C/T**	**T/T**	
Age (years)		47 (25-63)	45 (23-62)	41 (29-63)	0.65
Males (%)		74	63	43	0.15
DM (%)		10	8	14	0.88
dp-ucMGP (pmol/L)		1315 (755-2737)	1191 (650-2478)	1329 (799-1998)	0.57
MGP mRNA (RQ)≠		1.2 (0.4-3.1)	0.9 (0.4-1.8)	0.6 (0.5-0.8)	0.16
CAC score (AU) #		15 (0-1312)	0 (0-990)	0 (0-12)	0.06
Media VC score, (%)¤	0	15	15	29	0.05
	1	35	62	57	
	2	33	15	0	
	3	17	9	14	
rs1800802					
		**A/A**	**A/G**	**G/G**	
Age (years)		45 (23-62)	47 (25-64)	49 (36-60)	0.59
Males (%)		70	64	70	0.85
DM (%)		9	12	0	0.62
dp-ucMGP (pmol/L)		1323 (766-2526)	1232 (654-2016)	1121 (848-1884)	0.78
MGP mRNA (RQ)≠		1.0 (0.5-2.1)	1.3 (0.3-2.0)	1.3 (0.9-1.6)	0.88
CAC score (AU) #		0 (0-1061)	12 (0-1021)	15 (14-1710)	0.27
Media VC score, (%)¤	0	17	16	0	0.35
	1	54	39	40	
	2	15	34	40	
	3	14	10	20	

Data presented as median (range of 10th–90th percentile) or percentage. Differences between genotype groups were evaluated with Kruskal-Wallis and Pearson's Chi-square tests.

≠n=41; #n=91; ¤ n=108
